# Maternal and fetal thyroid dysfunction following porcine reproductive and respiratory syndrome virus2 infection

**DOI:** 10.1186/s13567-020-00772-2

**Published:** 2020-03-30

**Authors:** J. Alex Pasternak, Daniel J. MacPhee, John C. S. Harding

**Affiliations:** 1 Department of Large Animal Clinical Sciences, Western College of Veterinary Medicine, University of Saskatchewan, 52 Campus Dr, Saskatoon, SK S7N 5B4 Canada; 2 Department of Veterinary Biomedical Sciences, Western College of Veterinary Medicine, University of Saskatchewan, 52 Campus Dr, Saskatoon, SK S7N 5B4 Canada; 3 Present Address: Department of Animal Sciences, Purdue University, 270 S. Russell St, West Lafayette, IN 47907 USA

## Abstract

To better understand the host response to porcine reproductive and respiratory virus-2 (PRRSV2) we evaluated circulating thyroid hormone and associated gene expression in a late gestation challenge model. Pregnant gilts were inoculated at gestation day 85 and fetal samples collected at either 12 or 21 days post-infection (dpi). A subset of fetuses was selected for analysis based on viability and viral load categorized as either uninfected-viable (UNIF), high viral load viable (HV-VIA) or high viral load meconium stained (HV-MEC) and were compared with gestational age matched controls (CON). In dams, circulating levels of total T3 and T4 decreased in the acute period following infection and rebounded by 21 dpi. A similar effect was observed in fetuses, but was largely restricted to HV-VIA and HV-MEC, with minimal decrease noted in UNIF relative to CON at 21 dpi. Gene expression in fetal heart at 12 dpi showed significant decompensatory transcription of thyroid hormone transporters (SLC16A2) and deiodinases (DIO2, DIO3), which was not observed in brain. Correspondingly, genes associated with cell cycle progression (CDK1,2,4) were downregulated in only the heart of highly infected fetuses, while expression of their inhibitor (CDKN1A) was upregulated in both tissues. Finally, expression of genes associated with cardiac stress including CAMKD and AGT were upregulated in the hearts of highly infected fetuses, and a shift in expression of MYH6 to MYH7 was observed in HV-MEC fetuses specifically. Collectively, the results suggest PRRSV2 infection causes a hypothyroid state that disproportionally impacts the fetal heart over the brain.

## Introduction

Porcine reproductive and respiratory virus (PRRSV) readily crosses the normally restrictive epitheliochorial placenta of the pig during late gestation. Once infected, the fetus is capable of mounting a robust immune response [1, 2]. Even with this response, PRRSV strains which typically cause limited clinical signs or pathology in the dam may cause high levels of fetal mortality [3]. Given the inability to completely control or eradicate PRRSV2 though classical methods such as vaccination and biosecurity, there is continued interest in identifying the precise cause of fetal mortality following infection to facilitate development of alternative methods of control and potentially novel therapeutic treatments. With mounting evidence that viral damage to the placenta is not responsible for fetal compromise [4], there is renewed interest in the direct impact of this virus on the fetus and the potential longer-term implications of disruptions in the critical systems and processes that drive development and maturation of surviving fetuses.

Thyroid hormones are perhaps most widely known for their role in regulating the resting metabolic rate [5] and controlling appetite [6]. This group of hormones has a common structure with a variable number of iodine molecules and includes the highly abundant thyroxine (T4), its more bioactive derivative triiodothyronine (T3), and further metabolites such as reverse T3 (rT3) and 3,3 or 3,5 Diiodothyronines (T2). In the developing fetus, the bioactive forms of this hormone play additional roles in driving the accumulation of fetal mass and inducing maturation of critical tissues such as the brain and heart [7]. Unlike other mammals in which the fetus depends in part on maternal thyroid hormone production, high deiodinase activity of the porcine placenta acts as an enzymatic barrier to maternal thyroid hormone [8]. As a result, the thyroid gland of the fetal pig becomes active early in gestation and under normal conditions produces progressively higher levels of hormone leading up to parturition [9].

Under normal conditions, production of T4 and T3 is tightly regulated by homeostatic mechanisms in the hypothalamic-pituitary-thyroid axis (HPT). However, in response to severe illness the thyroid hormone system can be dysregulated in what is typically referred to as either non-thyroidal illness syndrome (NTIS) or euthyroid sick syndrome. Under such conditions, the normally homeostatic regulation of the HPT becomes allostatic and the altered set points render otherwise euthyroid individuals, systemically hypothyroid [10]. The altered regulatory setpoint leads to large-scale decreases in total circulating T3 and T4, often with no change or even decreased levels of thyroid stimulating hormone [11]. Like most mammals, the vital requirement for thyroid hormone in the developing pig fetus was established long ago [12], and as such, the development of an NTIS-like condition would be assumed to have a detrimental impact on development and possibly survival. In addition, previous work utilizing the model has clearly demonstrated a relationship between fetal susceptibility to infection, and fetal size or intrauterine growth retardation status which may be the related to thyroid function [13, 14].

While severe PRRSV-induced reproductive failure culminates in early farrowing, abortion, fetal death and mummification, the mechanisms underlying these losses are unknown and have been the focus of large-scale pregnant gilt challenge studies undertaken by our research team since 2012. One important discovery stemming from this research was the reduction in fetal growth rate following infection in the third trimester [15] which could be mediated by changes in thyroid hormones. Using the vast sample archive, we investigated (and herein report) the circulating levels of total T4 and T3 in the pregnant gilt following late gestation infection with a well characterized strain of PRRSV2. We then investigated changes in these hormones in fetuses derived from two well characterized, large-scale trials terminating at 12 and 21 days post maternal infection (dpi) [1, 13, 16]. To determine the association between thyroid hormone disruption and fetal viability, we utilized infection status as determined by viral load in multiple fetal tissues, and meconium staining as a marker of severe fetal pathology and imminent compromise [17, 18]. Finally, we examined the consequences of thyroid hormone disruption in the form of altered gene expression in multiple associated pathways within the fetal heart and brain.

## Materials and methods

### Animal model

Samples were derived from two large-scale, challenge experiments [16], for which highly detailed methodology has previously been published [3, 14]. In short, Landrace gilts bred to Yorkshire boars from a PRRSV-free nucleus herd were housed in one of two level II containment facilities at the University of Saskatchewan. In experiment 1, 111 gilts housed at the Vaccine and Infectious Disease Organization (VIDO) were challenged with 1 × 10^5^ TCID_50_ of PRRSV2 strain NVSL 97-7895 delivered 50/50 intramuscularly/intranasally at day 85 of gestation. Nineteen gestational-age-matched gilts housed at the Western College of Veterinary Medicine (WCVM) as uninfected controls were sham-challenged. All experiment 1 gilts were humanely euthanized at 21 days post maternal infection (21 dpi). In experiment 2, 31 gilts were challenged in an identical manner to those in experiment 1, with seven additional animals serving as uninfected controls with euthanasia undertaken at 12 dpi. Following removal, the gravid uterus was linearized and carefully dissected from each ovarian tip to the cervix to conserve fetal and placental pairings. Fetuses which appeared developmentally normal and exhibiting blood pulsations within the umbilical cord were categorized as live at the point of euthanasia. Live fetuses were then further categorized as viable (VIA; fetuses with normal skin colouring) and those showing evidence of meconium staining (MEC; fetuses with variable amounts of inspissated yellow–brown material on the face and body). Fetal blood was collected from the axillary artery and the serum separated and stored at −80 °C. From each fetus, the cervical and thoracic thymus was collected. In experiment 2, fetal brain and heart were also collected along with three anatomically unique sections of placenta and endometrium from: (i) immediately adjacent to the umbilical attachments, (ii) 10–15 cm from the umbilicus toward the anti-mesometrial side, and (iii) 10–15 cm from the umbilicus toward the ovary). All tissues were individually frozen at −80 °C for later analysis. Animal work was conducted in strict accordance with the guidelines of the Canadian Council of Animal Care and with approval of the University of Saskatchewan’s Animal Research Ethics Board (Exp. 1 Protocol # 20110102, Exp. 2 Protocol # 20160023).

### Viral load and group selection

PRRSV2 RNA concentration (target copies/µL or mg) was assessed in fetal serum and thymus as previously described in explicit detail [15]. In short, total RNA was extracted from a fixed volume or weight of either fetal sera or homogenized tissue using a QIAamp Viral RNA and RNeasy isolation kit, respectively (Qiagen, Hilden Germany). A one-step qPCR using a PRRSV2 specific primer–probe pair [19] was carried out in duplicate with quantification relative to a standard curve comprised of linearized plasmid containing the targeted region of ORF7. For Experiment 2, in addition to fetal serum and thymus, the three placental samples (separated from the endometrium prior to freezing) were collected from each fetus, subjected to weight normalized RNA lysis, and then pooled for RNA extraction and quantification. The resulting data on viral load was used in combination with meconium staining as a viability marker to identify subsets of fetal samples from the larger population in each experiment to represent biologically distinct, but consistent groups. Resistant fetuses were identified as those that were VIA and remained entirely uninfected (UNIF) having no detectable viral RNA in either serum or thymus, and in experiment 2 were also found to be placental PRRSV2 negative. Highly infected fetuses in Experiment 1 were selected with a cut off of > 5 log in serum and thymus and in Experiment 2 as > 4.5 log in serum, thymus and placenta, with the decreased threshold to account for shorter viral replication periods when terminated at 12 dpi. The highly infected fetuses from both trials were then subdivided into resilient fetuses [13] that remained VIA despite high viral load (HV-VIA) and susceptible fetuses who exhibited both high viral load and meconium staining (HV-MEC), an early marker of fetal compromise. For reference, a small number of control (CON) fetuses were drawn from the litters of gestation day matched, mock-inoculated gilts.

### Serum total T3 and T4

The concentration of total triiodothyronine (T3) and total thyroxine (T4) in maternal and fetal serum were determined using commercially available RIAs (MP Biomedical, Irvine, CA, USA) for each analyte, which have previously been used with porcine samples [20]. Assays were conducted in duplicate in accordance with the manufacturer’s directions with the exception that both samples and standards were diluted 1 in 4 prior to T4 assay to effectively reduce sample viscosity and thereby improve assay performance. Mean inter-assay variability across both experiments was found to be 9.6% and 10.6% for T3 and T4, respectively, as determined from three unique pools of porcine serum previously found to have high, medium and low thyroid hormone levels. Intra-assay variability across all samples was found to be 4.1% and 6.0% for T3 and T4, respectively.

### Genes expression analysis

Samples of heart and brain tissue from select fetuses were ground to a fine powder in a mortar and pestle under liquid nitrogen and total RNA isolated using Trizol (Thermofisher Scientific, Waltham, MA, USA) and a double precipitation method. RNA quantity was determined using a Nanodrop spectrophotometer (Thermofisher Scientific) and integrity was assessed using denaturing agarose gel electrophoresis [21]. Reverse transcription was carried out on 2 μg RNA using the High Capacity cDNA Reverse Transcription kit (Thermofisher Scientific). Gene-specific primers were designed or modified to correspond with current RefSeq mRNA sequences covering all predicted transcript variants (Table 1) for a series of housekeeping genes along with 21 genes of interest across three physiological processes. Where possible, primers were positioned to span exon–exon junctions, identified by the BLAST-like alignment tool (BLAT) against the Sus Scrofa 11.1 genome assembly. Primer efficiency for each target was determined to be greater than 90% and melting curve analysis suggested a single amplicon product. Real time PCR was carried out in duplicate on 20 ng cDNA using the SsoFast EvaGreen Supermix) and CFX qPCR system (BioRad, Hercules, CA, USA). Analysis of presumptive housekeeping genes identified STX5 and PRL19 as the most stable genes in the heart and HRPT, IPO8 and YWHAZ as the most stable in the brain. The geometric mean of each set of housekeeping genes was then used to normalize expression data within the respective tissue. The resulting expression data is expressed in the form of fold changes relative to the average expression of the CON group using the 2^−ΔΔCT^ method and fold changes reported based on the average of the group in question.

**Table 1:** **Gene targets and primer sequences used for qPCR**

	Official symbol	Gene ID	Forward primer	Reverse primer	Annealing temp (°C)	Amplicon length (bp)
House keeping	HPRT1	397351	5′-GGACTTGAATCATGTTTGTG-3′	5′-CAGATGTTTCCAAACTCAAC-3′	61	91
	IPO8	100511694	5′-AGGACAGTGGCAGAAGCAAA-3′	5′-TTCAGTTGTTGGTGGGCATA-3′	60	112
	RPL19	396989	5′-AACTCCCGTCAGCAGATCC-3′	5′-AGTACCCTTCCGCTTACCG-3′	60	147
	STX5	100628048	5′-TGCAGAGTCGTCAGAATGGA-3′	5′-CCAGGATTGTCAGCTTCTCC-3′	60	144
	YWHAZ	780440	5′-TGATGATAAGAAAGGGATTGTGG-3′	5′-GTTCAGCAATGGCTTCATCA-3′	62	203
Thyroid hormone reception	DIO2	414379	5′-CTCGGTCATTCTCCTCAAGC-3′	5′-TCACCTGTTTGTAGGCATCG-3′	61	140
	DIO3	414378	5′-CCTATCTGCGTGTCTGACGA-3′	5′-GCCTGCTTGAAGAAATCCAG-3′	61	92
	ITGAV	397285	5′-GCAACAGTGAAGCCTTAGCA-3′	5′-GCACACTGAAACGAAGACCA-3′	61	132
	ITGB3	397063	5′-AATGGGACACAGCCAACAAT-3′	5′-CCACAATCCTGGGACAGTCT-3′	61	126
	SLC16A10	100513770	5′-CACCCATTGCAGGGTTACTC-3′	5′-TATGGAGCCAAGGGATGAAA-3′	61	117
	SLC16A2	100513513	5′-AGTGGAGTTCCAAGCAGCAT-3′	5′-AGCCCAAACGATCAGTGAAT-3′	61	95
	THRA	397387	5′-GAGGAGAACAGTGCCAGGTC-3′	5′-CGACACACTGCTCGTCTTTG-3′	61	121
	THRB	396776	5′-AAGGCTGCAAGGGTTTCTTT-3′	5′-TGGCACTGATTTCTGGTGAC-3′	61	112
Cell cycle progression	CCND1	100738589	5′-TGTGCCACAGACGTGAAGTT-3′	5′-GGTGGTAGGACAGGAAGCTG-3′	61	118
	CDK1	100155762	5′-CAGCTCGCTACTCAACTCCA-3′	5′-GAGTGCCCAAAGCTCTGAAA-3′	61	135
	CDK2	100154715	5′-CGGAGCTTGTTATCGCAAAT-3′	5′-AGGGGTAGGGTTCACAAAGG-3′	61	143
	CDK4	100144492	5′-TGGTTACAAGTGGTGGGACA-3′	5′-CCACAGAAGAGAGGCTTTCG-3′	61	208
	CDKN1A	100152215	5′-CATGTGGACCTGTTGCTGTC-3′	5′-TTAGGGCTTCCTCTTGGAGA-3′	61	168
Cardiac development and function	AGT	100157073	5′-GCACTTCCAAGGAAAGGTGA-3′	5′-CGACACTGAGGTGGTGTTGT-3′	60	82
	ATP2A2	396875	5′-AACGCCCTCAACAGTTTGTC-3′	5′-AGCCACTGGGTCAAATTCAG-3′	61	173
	CAMK2D	397674	5′-CAGTACCCATCAAGCCATCC-3′	5′-TGCATGAAGAGGAGGAGAGG-3′	60	197
	MYH6	100736765	5′-CTTCGGGAAATTCATCAGGA-3′	5′-GCTCTGGCTTCTTGTTGGAC-3′	62	161
	MYH7	396860	5′-ATCCACCCAAGTTCGACAAG-3′	5′-AGATCATCCAGGAGGCGTAG-3′	62	105
	PLN	397421	5′-ACCATTGAAATGCCTCAACA-3′	5′-CGATGATGCAAATCAGCAAG-3′	61	97
	RYR2	396856	5′-GTGGATACCAGCCAGATCGT-3′	5′-CATACTGCCAGCCAAGTTCA-3′	60	115

### Statistical analyses

Data processing and analyses were performed in R 3.6.1 [22]. Statistical analysis of thyroid hormone concentration in maternal serum was conducted using a linear mixed model from the nlme package with a random slope and intercept model to account for repeat measures [23]. Statistical analysis of fetal thyroid hormone levels in both experiments was carried out using a one-way ANOVA. For both maternal and fetal thyroid hormones, pairwise contrasts among group were generated using the emmeans package [24] with Dunn-Sidak’s correction to account for multiple comparisons and the results reported in SI units (nmol/L) ± standard deviation. Gene expression data was found to be largely non-normal and was therefore assessed for all genes using a consistent non-parametric methodology consisting of Kruskal–Wallis test followed by post hoc pairwise comparisons via the Wilcoxon rank sum test with the resulting *P* value adjusted for multiple comparisons using the Bonferroni correction. Data was visualized using the ggplot2 package [25] with observed statistical differences (*P* < 0.05), where present, marked with unique superscripts.

## Results

### Thyroid hormone response in maternal serum

**Figure 1:**
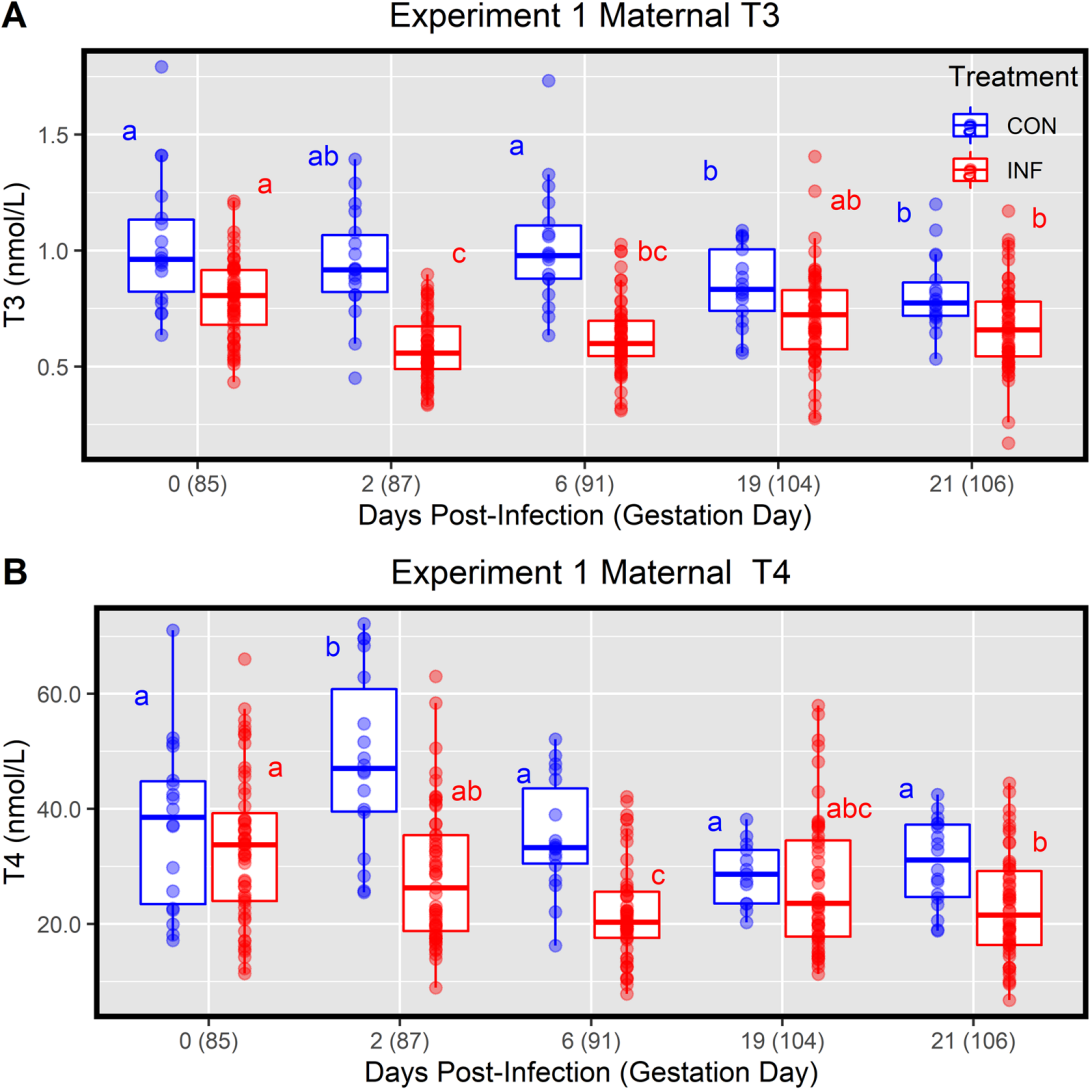
**Thyroid hormone levels in pregnant gilt sera over time**. Sera from PRRSV2 challenged gilts (INF *n* = 57) from 0 to 21 days post-inoculation and gestational day matched, non-inoculated control gilts (CON *n* = 18). Total triiodothyronine (**A**) and thyroxin (**B**) were measured via RIA with data is presented as a standard boxplot with unique superscripts denote within group statistical differences (*P *< 0.05) over time.

To determine the impact of PRRSV2 on regulation of the thyroid hormone system, we first evaluated total T3 and T4 in serum from pregnant gilts (*n* = 57) over a 21-day window following infection at day 85 of gestation. Prior to challenge, the PRRSV-infected gilts had a baseline mean serum T3 and T4 of 0.79 ± 0.17 nmol/L and 32.80 ± 12.96 nmol/L, respectively (Figure 1). Following infection, serum T3 levels in these gilts were significantly depressed at dpi 2 (0.58 ± 0.14 nmol/L, *P* < 0.001) and 6 (0.63 ± 0.16 nmol/L, *P* < 0.001) relative to 0 dpi. Levels of T3 rebounded thereafter, showing no significant difference relative to baseline at 19 dpi (0.71 ± 0.21 nmol/L, *P* = 0.109), however, 21 dpi levels were again found to be significantly lower (0.67 ± 0.19 nmol/L, *P* = 0.109) relative to 0 dpi levels. Total T4 was similarly impacted, with a significant decrease relative to 0 dpi found at 6 (22.16 ± 8.55 nmol/L, *P* < 0.001) and 21 dpi (22.99 ± 9.03 nmol/L, *P* < 0.001), and a trend toward significance at 19 dpi (27.5 ± 13.46 nmol/L, *P* = 0.099).

To determine if the observed effect in either analyte was simply the result of healthy endocrine regulation during late gestation, we next evaluated the concentration of these hormones in the gestational aged-matched controls. Levels of T4 at 0 dpi (day 85 of gestation) did not differ significantly from those of the PRRSV-infected group (37.19 ± 14.58 nmol/L, *P* = 1.000), however, levels of T3 (1.03 ± 0.29 nmol/L, *P* = 0.003) were significantly elevated; likely the result of the alternative housing conditions under which uninfected control gilts were housed, so all subsequent analysis was only conducted within group. However, unlike the immediate decrease observed in the infected gilts post-challenge, no significant change in T3 levels was observed in the control group at 2 (0.94 ± 0.23 nmol/L, *P* = 0.684) and 6 (1.02 ± 0.26, *P* = 1.000) dpi relative to 0 dpi (85 days gestation). Levels of T3 in the control group did show a significant decrease at 19 (0.85 ± 0.17 nmol/L, *P* = 0.018) and 21 (0.81 ± 0.16 nmol/L, *P* < 0.001) following mock inoculation. The concentration of T4 in control animals showed no significant changes from the initial value, except for a significant elevation at 2 dpi (gestation day 87) (48.43 ± 15.42 nmol/L, *P* = 0.010) relative to 0 dpi (Figure 1).

### Thyroid hormone response in fetal serum

**Figure 2:**
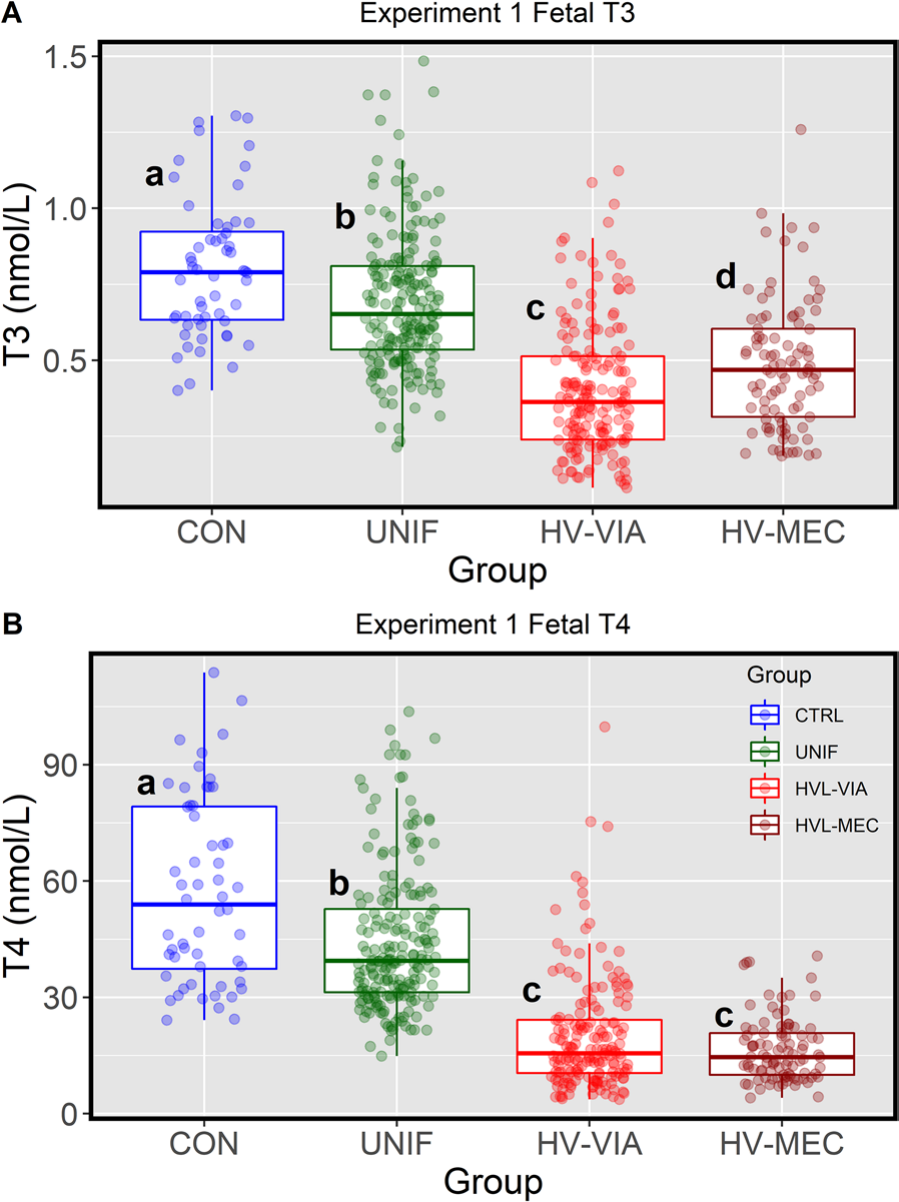
**Thyroid hormone levels in sera from fetuses at 21** **days post-infection**. Sera was derived from gestational day matched, non-inoculated control gilts (CON *n* = 56) and from PRRSV2 challenged dams at 21 days post-inoculation (Experiment 1) and classified based on serum and thymic viral load as uninfected (UNIF *n* = 201), high viral load viable (HV-VIA *n* = 171) or high viral load meconium stained (HV-MEC *n* = 93). Total triiodothyronine (**A**) and thyroxin (**B**) were measured via RIA with data is presented as a standard boxplot with unique superscripts denote statistical differences (*P *< 0.05).

We next examined the impact of PRRSV2 on the regulation of fetal thyroid hormone levels in the context of PRRS resilience indicated by fetal viral load and fetal preservation (viability versus meconium staining) in experiment 1 samples collected at 21 days after maternal inoculation (Figure 2). At this stage of gestation, CON fetuses had an average serum T3 and T4 of 0.80 ± 0.23 nmol/L and 57.25 ± 23.84 nmol/L, respectively. Relative to CON, the UNIF fetuses showed a significant decrease in the level of both T3 (0.69 ± 0.22 nmol/L, *P* = 0.005) and T4 (44.43 ± 18.38 nmol/L, *P* < 0.001). This disruption was even more severe in both fetal groups with high viral load (HV-VIA and HV-MEC) where the levels of both hormones were significantly (*P* < 0.001) below that of both CON and UNIF groups. Interestingly, while the level of T4 did not differ between highly infected fetal groups (HV-VIA = 20.11 ± 14.74 nmol/L and HV-MEC = 16.51 ± 8.25 nmol/L, *P* = 0.332), the levels of T3 were significantly higher (*P* = 0.0267) in HV-MEC (0.49 ± 0.21 nmol/L) than HV-VIA (0.41 ± 0.22 nmol/L).

**Figure 3:**
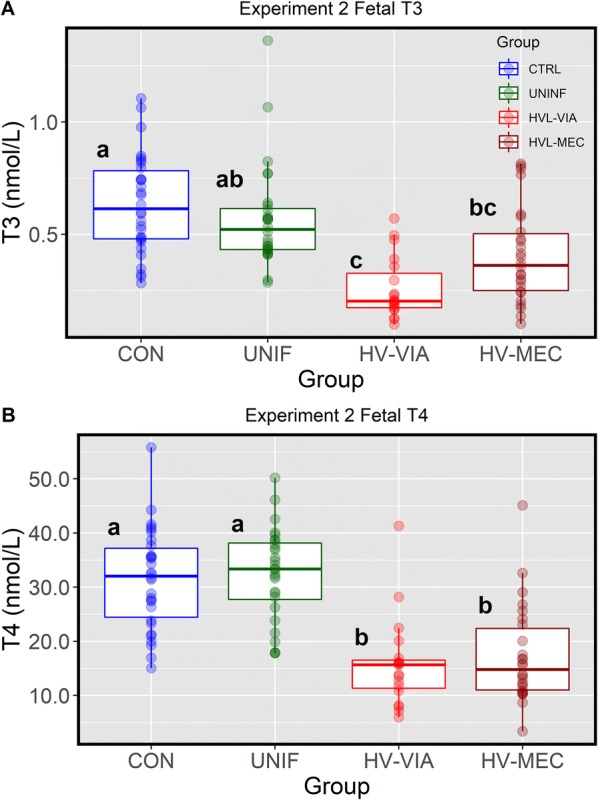
**Thyroid hormone levels in sera from fetuses at 12** **days post-infection**. Sera derived from gestational day matched, non-inoculated control gilts (CON *n* = 30) and from PRRSV2 challenged dams at 12 days post-inoculation (Experiment 2) and classified based on serum, thymic and placental viral load as uninfected (UNIF *n* = 28), high viral load viable (HV-VIA *n* = 19) or high viral load meconium stained (HV-MEC *n* = 26). Total triiodothyronine (**A**) and thyroxin (**B**) were measured via RIA with data is presented as a standard boxplot with unique superscripts denote statistical differences (*P *< 0.05).

To determine if this effect was present in fetuses at earlier point of infection, T3 and T4 were assessed in serum samples from a second large scale D85 maternal infection trial (Experiment 2) where gilts were terminated at 12 dpi or 97 days of gestation (Figure 3). At this earlier stage of gestation, both fetal hormones were found to be less abundant than at 21 dpi (gestation day 106), with average T3 and T4 found to be 0.63 ± 0.22 nmol/L and 31.45 ± 9.2 nmol/L, respectively, among CON and no significant difference from these values was found in fetuses classified as UNIF. Similar to that observed in experiment 1, both highly infected fetal groups (HV-VIA and HV-MEC) showed significant depression relative to both UNIF and CON. No significant difference in T4 was found between these groups (HV-VIA = 15.82 ± 8.23 nmol/L and HV-MEC = 17.31 ± 9.00 nmol/L, *P* = 0.943), however similar to the effect observed in Experiment 1, a trend (*P* = 0.086) toward increased T3 in HV-MEC (0.41 ± 0.21 nmol/L) relative to HV-VIA (0.26 ± 0.14 nmol/L) was identified.

### Expression of genes associated with peripheral reception of thyroid hormone

**Figure 4:**
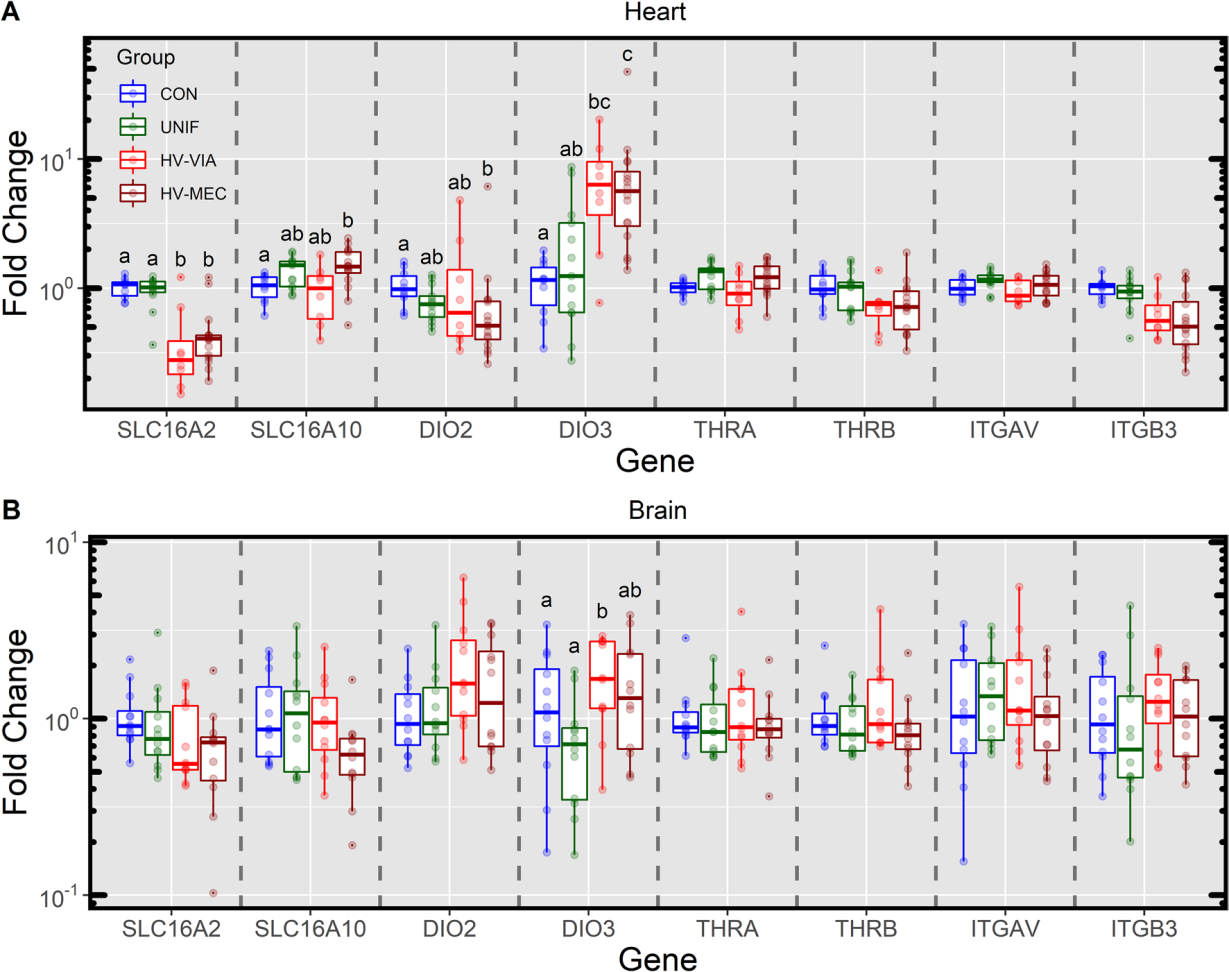
**Fetal gene expression associated with peripheral thyroid hormone reception**. Genes associated with uptake (SLC16A2 & SLC16A10), modification (DIO2 & DIO3) and reception (THRA, THRB, ITGAV &ITGB3) of thyroid hormone in the heart (**A**) and brain (**B**) tissue of fetuses derived from gestational day matched, non-inoculated control gilts (CON *n* = 10 heart and 12 brain) and from PRRSV2 challenged dams at 12 days post-inoculation and classified based on serum and thymic viral load as uninfected (UNIF *n* = 13 heart and 12 brain), high viral load viable (HV-VIA *n* = 8 heart and 12 brain) or high viral load meconium stained (HV-MEC *n* = 16 heart and 12 brain). Gene expression was determined by qPCR and fold changes were calculated relative to the average of the CON with unique superscripts denoting statistical differences (*P *< 0.05) within a given tissue and gene.

The biological action of thyroid hormones can be controlled at the cellular level through uptake, metabolism and receptor activity. To better understand how these factors were altered in peripheral tissues of the fetus in response to the observed systemic dysregulation in hormone levels, we next evaluated the expression of select genes in the heart and brain collected at 12 dpi. Expression of genes associated with transmembrane transport of thyroid hormone (SLC16A2 and SLC16A10) and peripheral deiodinase enzymes (DIO2 and DIO3) showed significant alteration in the fetal heart (Figure 4A). More specifically, the expression of SLC16A2 was reduced relative to CON in both HV-VIA (-3.1 fold, *P* = 0.040) and HV-MEC (−2.4 fold, *P* = 0.005). A minor upregulation in SLC16A10 (1.4 fold, *P* = 0.040) was found in HV-MEC relative to CON. The outer ring deiodinase DIO2 was slightly down regulated (−1.7 fold *P* = 0.017) in HV-MEC again relative to CON. Finally, the inner ring deiodinase DIO3 was significantly upregulated relative to CON in both HV-VIA (5.2 fold, *P* = 0.031) and HV-MEC (5.2, *P* < 0.001) fetuses. No significant differences were observed in cardiac expression of either nuclear (THRα and THRβ) or extracellular (ITGAV and ITGB3) receptors, though ITGB3 did show a trend toward reduced expression in both HV-VIA (−1.65 fold, *P* = 0.087) and HV-MEC (−1.85 fold, *P* = 0.063) relative to CON. Unlike the heart, fetal brain (Figure 4B) showed almost no significant alterations in the expression of the genes in this pathway with the singular exception of a small (2.4 fold) but significant (*P* = 0.031) decrease in DIO3 in UNIF fetuses relative to HV-VIA.

### Expression of genes associated with regulation of cell cycle progression

**Figure 5:**
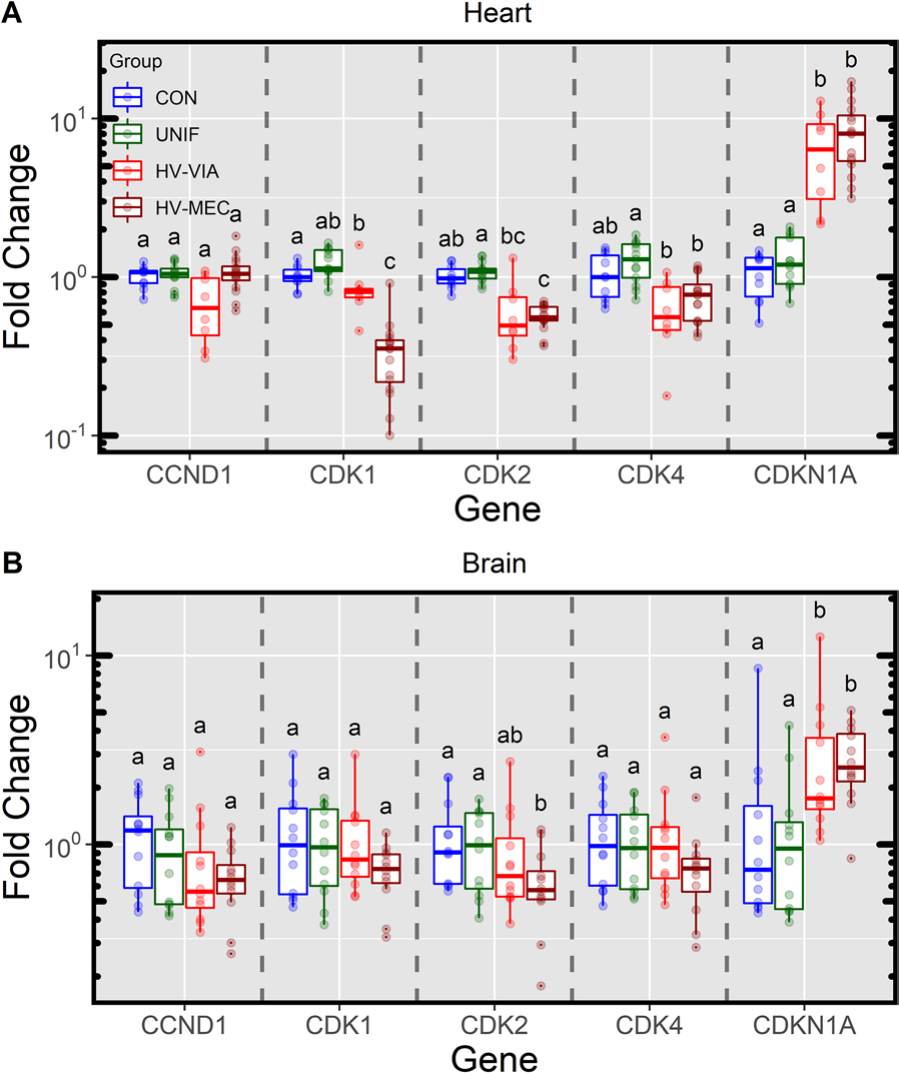
**Fetal gene expression associated with regulation of cell cycle progression**. Expression of genes responsible for promoting cell cycle progression (CCND1, CDK1, CDK2 and CDK4) and their inhibitor (CDKN1A) in the heart (**A**) and brain (**B**) tissue of fetuses derived from gestational day matched, non-inoculated control gilts (CON *n* = 10 heart and 12 brain) and from PRRSV2 challenged dams at 12 days post-inoculation and classified based on serum and thymic viral load as uninfected (UNIF *n* = 13 heart and 12 brain), high viral load viable (HV-VIA *n* = 8 heart and 12 brain) or high viral load meconium stained (HV-MEC *n* = 16 heart and 12 brain). Gene expression was determined by qPCR and fold changes were calculated relative to the average of the CON with unique superscripts denoting statistical differences (*P *< 0.05) within a given tissue and gene.

Given the established role of thyroid hormones in regulating cell cycle progression, we next investigated the expression of five genes of interest which are involved in controlling the cell cycle in both the fetal heart and brain (Figure 5). This included a set of Cyclin dependant kinases which collectively cover all stages of the cell cycle, with CDK1 promoting S to G2 and G2 to M, while CDK4 and CDK2 drive M to G1 and G1 to S, respectively. CDKN1A (previously known as P21) is perhaps the most well studied gene in this pathway, and is known to act as an inhibitor of all three CDKs evaluated. In both tissues, expression did not differ between UNIF and CON fetuses for any of the 5 genes assessed. Among the cyclin dependant kinases, no significant differences were found between HV-VIA fetuses and CON, however, trends toward downregulation were noted for CDK2 (−1.8 fold, *P* = 0.067). A number of significant differences were, however, noted between the HV-VIA and UNIF groups, likely owing to the small numerical increase in fetuses sampled in the latter over CON. HV-MEC fetuses showed decreased expression of CDK1 (−3.3 fold, *P* = 0.026) and CDK2 (−1.8 fold, *P* < 0.001) relative to CON. The most significant alteration in cardiac expression as it relates to cell cycle progression was identified in the inhibitor CDKN1A that was upregulated relative to CON in both HV-VIA (5.49 fold, *P* = 0.002) and HV-MEC (7.48 fold, *P* < 0.001). Interestingly, this was the only gene in this pathway found to be dysregulated in the brain, albeit to a lesser extent, with a significant upregulation over CON in HV-MEC (2.6 fold, *P* = 0.031) and a trend toward significance in HV-VIA (2.35 fold, *P* = 0.099).

### Alterations to fetal cardiac gene expression

**Figure 6:**
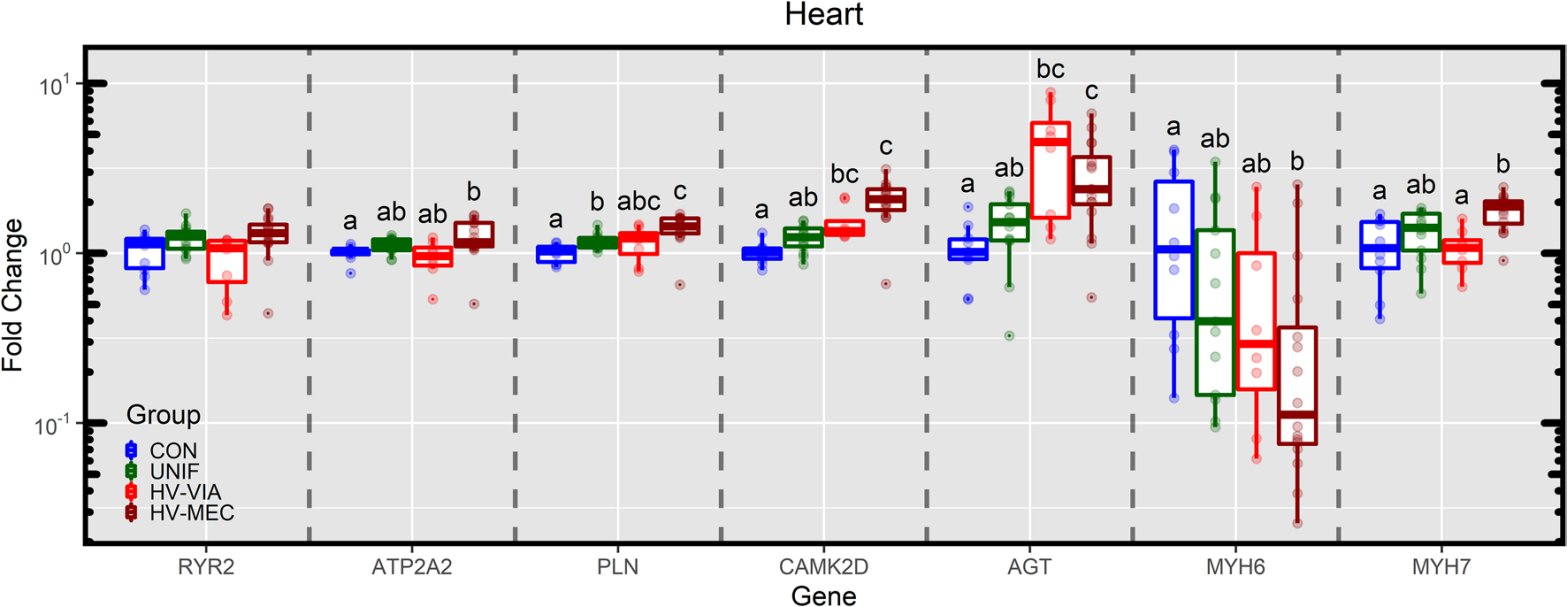
**Fetal gene expression associated with cardiac function**. Expression of genes associated with cardiac contractility (RYR2, ATP2A2 and PLN), response to stress (CAMK2D and AGT), and muscle fiber maturation (MYH6 and MYH7) in the heart of fetuses derived from gestational day matched, non-inoculated control gilts (CON *n* = 10) and from PRRSV2 challenged dams at 12 days post-inoculation and classified based on serum and thymic viral load as uninfected (UNIF *n* = 13), high viral load viable (HV-VIA *n* = 8) or high viral load meconium stained (HV-MEC *n* = 16). Gene expression was determined by qPCR and fold changes were calculated relative to the average of the CON with unique superscripts denoting statistical differences (*P *< 0.05) within a given gene.

To better understand the impact of infection and the resulting dysregulation in thyroid hormone on the fetuses, we next focused our attention on the expression of a series of genes associated with cardiac function and development (Figure 6). In the hearts of HV-MEC fetuses, small but significant upregulations in both ATP2A2 (1.2 fold, *P* = 0.010) and PLN (1.4 fold, *P* = 0.002) relative to CON were identified. The gene CAMK2D was found to be significantly upregulated over CON in both HV-VIA (1.5 fold, *P* = 0.007) and HV-MEC (1.9 fold, *P* = 0.002), with AGT showing similar upregulation (3.5 fold, *P* = 0.018 and 2.5 fold, *P* = 0.005 respectively). Cardiac myosin genes showed opposing changes in HV-MEC hearts relative to CON, with expression of MYH6 significantly downregulated (−5.5 fold, *P* = 0.034) and MYH7 upregulated (1.7 fold, *P* = 0.015).

## Discussion

As one of the more economically devastating endemic diseases impacting swine production, a great deal of research has been focused on understanding PRRSV and its interaction with its host. However, the mechanism by which it causes fetal death, a significant aspect of its economic impact [26], remains elusive. Thyroid hormones act to regulate numerous physiological mechanisms in a wide variety of peripheral tissues and are particularly important in regulating fetal development. The critical link between normal thyroid function and successful reproduction in swine was established prior to 1920, when iodine supplementation of maternal feed was found to alleviate the “hairless pig malady” which resulted in abnormal piglets that were either stillborn or died shortly after birth [27]. While significant advances in animal nutrition since this time have effectively eliminated micronutrient deficiencies, the susceptibility of the thyroid hormone pathway to disruption makes it an important area of study in the context of viral infections such as PRRSV. In this present study, we evaluated the impact of maternal and fetal PRRSV infection on thyroid endocrinology following challenge in third trimester.

Interpretation of the post-infection dynamics of maternal thyroid hormone levels is complicated by the observed pre-challenge differences in T3 in PRRSV-infected compared to the gestation age matched controls. Feed restriction for as little as 24 h has been shown to cause a significant decrease in the circulating T3 and T4 levels in swine [28]. There is also established interplay between feed intake and ambient temperatures [20, 29, 30] as well as additional alterations following adaptation to new diets [31]. Given that the control and infected gilts originated from the same genetic nucleus farm, the apparent susceptibility of the thyroid hormone system to environmental conditions leads us to posit that the observed pre-challenge difference likely stems from minor differences in the conditions between the facilities in which these two groups were housed. For this reason, our analysis focused on the within group temporal effect in maternal T3 and T4. With this limitation in mind, we find a significant decrease in both hormones in the acute period following infection at a stage in gestation where control animals show stable T3 and an increase in T4. Levels of both hormones appear to rebound toward day 21, a point where past research has shown viremia in these gilts has largely subsided [15]. This rebound occurs during a stage of gestation when maternal thyroid hormone levels appear to decrease, which would be consistent with previously established temporal patterns in pregnant swine [12]. Collectively these results indicate that PRRSV infection during late gestation causes significant dysregulation in the normal temporal dynamics of thyroid hormone in the pregnant pig.

Thyroid hormones play a critical role in fetal development, where in addition to regulating metabolic rate, they promote development and maturation of fetal organs and the overall accretion of fetal mass [7]. In order to support their exponential growth rate [32], thyroid hormone levels increase in the porcine fetus throughout gestation, surpassing maternal concentrations prior to parturition [9, 12]. This late gestation trend is evident in the present study, based on levels of both T3 and T4 which within each fetal group were higher at gestation day 106 (21 dpi; Experiment 1) compared to gestation day 97 (12 dpi; Experiment 2), further underscoring the importance of thyroid hormone in later term fetal development. Within the context of this increased fetal requirement, we observed a significant decrease in thyroid hormone associated with fetal PRRSV infection, regardless of the sample collection timing relative to maternal infection. This effect appears largely compartmentalized to the highly infected fetuses, with UNIF fetuses only suppressed relative to CON at 21 dpi. This is in contrast to the observations made during the study of the hairless pig malady, that showed congenital hypothyroidism would produce goitrous like increase in thyroid weight birth [27]. Although not specifically evaluated during either study reported herein, no gross alterations in the size of the fetal thyroid gland were noted during removal of the fetal cervical thymus, which lies in close proximity, perhaps suggesting an alternative mechanism. Previous assessment of the fetal immune response at 21 dpi did identify small but significant upregulation in a subset of cytokines in a similar population of fetuses with no detectable PRRSV viral load [1] suggesting either infection below the detection limit of our qPCR assay or an indirect impact of maternal infection. The lack of difference in the thyroid hormone levels of UNIF fetuses at 12 dpi may result from the reduced statistical power inherent to this smaller experiment, or an insufficient time interval for the impact of maternal infection to indirectly influence the fetus.

In species with hemochorial placenta such as humans and rodents, maternal thyroid hormone is thought to complement fetal production, particularly under conditions of fetal hypothyroidism [7]. In contrast, the epitheliochorial placental of the pig actively deiodinates maternal thyroid hormone, which is thought to effectively isolate the fetus [8]. However, in one trial involving thyroidectomy and cannulation of the late gestation pig fetus, levels of T4 only dropped to 2.17 µg/dl following post-surgical recovery despite an established loss of thyrotropin releasing hormone (TRH) response [33]. This result, combined with the comparatively short half-life of T4 relative to the experiment, would suggest that like hemochorial species, the active barrier of the porcine placenta is either incomplete or may be modulated under conditions of severe fetal hypothyroidism to allow for transfer from the dam. In both Experiment 1 and 2, thyroid hormone in the HV-VIA fetus dropped to levels below that observed in the aforementioned thyroidectomy trial, which may be a result of the simultaneous depression in maternal and fetal thyroid hormone levels we observed. The significant changes in swine genetics and husbandry since the late 1980s make a direct comparison problematic, but it is nonetheless clear that the decrease in thyroid hormone levels in these PRRSV2 infected fetuses represents a significant dysregulation in the HPT roughly equivalent to thyroidectomy.

Other thyroidectomy experiments conducted in a variety of species have demonstrated that while such intrusion causes widespread physiological disruption, it is not typically terminal to the fetus [33–37]. Unlike these models, disruption of the thyroid hormone system in the present experiments was not restricted to the fetus, but coincident with maternal hypothyroidism making it similar to the hairless pig malady discussed previously. The endocrinological hallmarks of NTIS are poorly defined, with the impact of circulating levels of free and total T3 and T4, and the influence of TSH largely dependent on the specific underlying stressor or disease [10]. In the absence of data from a validated porcine TSH assay, it is difficult to compare the present results with more established examples of NTIS. In addition, the total T3 and T4 levels, which were assessed in this present research, are often the first and most drastically altered markers of NTIS [11]. As such, they are good indicators of disruption but poorly suited to distinguishing the degree of NTIS severity or the underlying pathophysiological mechanism.

Interestingly, T3 in the susceptible HV-MEC fetuses, while significantly depressed relative to CON, did not decrease to the same extent as that observed in the more resilient HV-VIA group. This rather surprising result may indicate that suppression of the fetal thyroid hormone system confers some benefit during serious viral infection. PRRSV infection has been shown to significantly increase both the occurrence of placental separation [4] and apoptosis [38]. These disruptions would compromise placental efficiency during a period of elevated fetal growth and requirement, creating a transport deficit likely to result in fetal hypoxia. A global reduction of metabolic rate resulting from low circulating thyroid hormone levels may help to lessen this deficit and thereby offset the physiological strain on the infected fetus. Such a conclusion would be consistent with models of thyroid allostasis where alterations to the endocrine system can be beneficial when demand for resources exceed supply, however, while such adjustments benefit acute survival, they often come at the cost of adverse effects over the longer term [10].

In the fetus, the consequence of such allostatic regulation is likely to come in the form of altered development and maturation of peripheral organs such as the heart [35, 39] and brain [36, 40] which are developmentally and functionally sensitive to thyroid hormone. As much of the response to thyroid hormones is dictated by the local uptake, metabolism and receptor availability, we hypothesised that the low circulating hormone concentrations in highly infected fetuses would result in compensatory up regulation within one or more of systems. Among the most critical of these is DIO2, a strictly outer ring deiodinase which primarily converts T4 to the more bioactive T3. The enzyme is typically found localized to the endoplasmic reticulum where it mediates entry of the resulting T3 into the nucleus where it can bind and activate nuclear receptors THRα and THRβ [41]. In juxtaposition to this, DIO3 is located on the plasma membrane where it catalyzes the inner ring deiodinations necessary to convert T4 to the inactive rT3, or T3 to a lower potency T2. Although this characterization of deiodinase activity is to some degree over simplified (see [42] for a complete review), the decrease in DIO2 and increase in DIO3 expression observed in hearts from HV-VIA and HV-MEC would be consistent with reduced bioactivity of thyroid hormone in the fetal heart. In addition, we observed a rather large downregulation in heart SLC16A2 (AKA MCT8) in highly infected fetuses, which is responsible for import of thyroid hormones into the cytoplasm [43]. Although some of this loss in transporter abundance would be counteracted by the minor upregulation in SLC16A10 (AKA MCT10) which possesses similar functionality, the collective pattern of gene expression observed in the heart would suggest a downregulation of its capacity to respond to thyroid hormone. In the context of reduced thyroid hormone levels observed in high viral load fetuses, this anti-homeostatic pattern of gene expression would serve to exacerbate the impact of hypothyroidism on the heart. In stark contrast to this, almost no alterations to these gene pathways, compensatory or otherwise, were seen in the brain suggesting the impact of systemic hypothyroidism on proliferation and development would be less severe in this tissue.

To evaluate the impact on brain and heart, we next examined genes associated with cell cycle progression, a pathway which is known to be regulated by nuclear thyroid hormone receptors [44, 45]. In both tissues CDKN1A, an inhibitor of cell cycle progression, was significantly upregulated. In the brain, the resulting suppression of the cell cycle is consistent with previous work of Antonson et al. [46] which showed decreased in total brain weight and cell number reductions in specific hippocampal regions of fetuses from PRRSV infected sows. Although the Antonson [46] study did not find significant differences in Ki67 + cell numbers in the brain, this may be the result of a failure to distinguish fetuses based on infection status [46] as our results clearly show the decrease in thyroid hormone and alteration in this gene pathway are restricted to infected fetuses. Consistent with the decompensation of the TH reception pathway in the heart discussed above, we found greater upregulation in CDKN1A in the heart compared to brain, along with downregulation of three associated pro-proliferation cyclin dependant kinases (CDK1, CDK2 and CDK4) among highly infected fetuses. We have recently shown that cell cycle progression in the thymus of PRRSV infected fetuses is similarly downregulated [1]. This comparatively large impact on cell cycle progression in non-neuronal tissue would further suggest a brain-sparing effect. This is consistent with the impact thyroidectomy in sheep which caused a decrease in total brain weight but to a lesser extent than the reduction in fetal weight [36]. It is worth noting that the impact of thyroid hormone on proliferation in the fetal heart is controversial. Early in vitro studies showed T3 dampened proliferative activity in fetal ovine cardiomyocytes [47], however, this result is somewhat contradicted by later fetal thyroidectomy studies in sheep which identified a reduction in total weight and a large downregulation in Ki67 staining in the heart in the absence of this endocrine system [35]. Our present research does not provide evidence for a direct link between reduced systemic T3/T4 and the suppression of cell cycle progression in the heart, however, the significant observation of anti-proliferative gene expression perhaps support the pleiotropic effect of thyroid hormones on proliferation [48].

Previous studies evaluating the histopathology in fetal hearts from the pregnant gilt model found heart lesions (mild, focal to multifocal, lymphocytic myocarditis and perivascular cuffing) were rarely observed in the fetal population (3.92% of PRRSV-infected fetuses) but were more prevalent in meconium stained and decomposed fetuses (14.5% and 5.9%, respectively) compared to viable fetuses (1%) providing some evidence that heart is involved in fetal compromise [4]. To further evaluate the impact of hypothyroidism on cardiac function, we investigated a combination of genes associated with contractile activity, response to stress and maturation for extrauterine life, all of which have previously been shown to be altered in the hypothyroid fetus, particularly in thyroidectomy models [35, 39, 49]. Interestingly, the majority of the genes assessed did not respond as expected to the hypothyroid state identified in the highly infected fetal groups (HV-VIA and HV-MEC). This response is particularly surprising in the context of the previously mentioned downregulation in cardiac expression of the response pathway. It is therefore of interest to evaluate the specific role of these genes in the fetal heart and their expected consequences on cardiac function and fetal viability.

Normal contractile function of the heart is dependent on the timely and coordinated movement of calcium between the cytosol and sarcoplasmic reticulum [50]. The flood of calcium from the sarcoplasm required for contraction passes through a channel created by protein expressed from the RYR2 gene, which is then reversed by active pump comprised of protein from the ATP2A2 gene (AKA SERCA2α) [51]. The activity of the ATP2A2 pump is regulated in part by PLN which acts as a potent inhibitor in its phosphorylated state [52]. Contractile activity of the myocardium is upregulated by thyroid hormones which have been shown to signal an increase expression of RYR2 and ATP2A2 [53, 54] and a decrease in PLN expression [55]. In the absence of thyroid hormone, thyroidectomized ovine fetuses showed a significant downregulation in cardiac ATP2A2 [35]. It is therefore surprising that expression of ATP2A2 and PLN was unchanged and slightly upregulated in the hypothyroid HV-VIA and HV-MEC fetuses, respectively. Collectively these results would suggest that another, as yet undefined, mechanism is capable of maintaining normal expression of these genes in the PRRSV infected fetuses.

The gene CAMK2D encodes one of the multifunctional calmodulin-dependent protein kinases and is the predominant member of this gene family expressed in the heart. Although this gene is constitutively expressed in the heart, upregulation of gene expression and the resulting increase in protein abundance in the myocardium has been associated with adverse outcomes for some time [56]. For example, in both diabetic humans and mice, cardiac up-regulation of CAMK2D has been associated with pathology including hypertrophy, fibrosis and apoptosis [57]. The role of this protein in cardiac pathology was more clearly established in a knockout mouse model where animals exhibited normal heart function but when stress was applied in the form of pressure overload, cardiac hypertrophy did not develop in the absence of CAMK2D [58]. These negative outcomes may stem from the ability of CAMK2D to initiate a cascade of inflammatory gene expression through activation of the NF-κB pathway [59]. Expression of this protein, albeit an altered form, has also been shown to arrest cell cycle progression [60] and thus, upregulation of the gene in the present study is not only an indicator of cardiac stress, but is also consistent with the previously discussed disruptions in cardiac cell cycle progression.

Another critical mechanism involved in the development of abnormal cardiac pathology is the renin-angiotensin system [61]. Canonically speaking, the precursor peptide for this system, angiotensinogen, is produced from the AGT gene in the liver and processed distally to produce the more biologically active angiotensin I and II. However, expression of the AGT gene along with the array of enzymatic factors and receptors required to create a localized renin-angiotensin response have been found to be expressed in the neonatal heart where it is thought to regulate contraction, growth and development [62]. Similar to CAMK2D, abnormal activation of the cardiac renin-angiotensin system is associated with cardiac hypertrophy [63]. Unlike the present study where we observed increased expression in the hypothyroid fetus, upregulation of this system in the heart is more commonly associated with a hyperthyroid state [61]. The expression of AGT in the heart may not be driven by thyroid hormones, as activation of this system can be induced through mechanical stretching of the myocytes and cardiac fibroblasts in a manner consistent with hypertrophy and pressure overload [63].

Contraction of the heart is primarily mediated by two specific isoforms of cardiac myosin, MYH6 (aka MHCα) and MYH7 (AKA MHCβ), with expression in the adult biased toward atria and ventricles, respectively [64]. The relative abundance of each isoform alters the mechanical properties of the heart and the MYH6:MYH7 ratio is known to change during fetal cardiac development and in the postnatal period [65]. In the hypothyroid HV-MEC fetuses, we observed a significant downregulation in expression of MYH6 and an upregulation in MYH7. This observed antithetical shift in expression of cardiac myosin is consistent with previous studies showing hypothyroidism induced chemically in the rat caused a similar downregulation in MYH6 (AKA α-myosin) and upregulation in HYH7 (AKA β-myosin) [66]. This observed shift in myosin isoform is an indicator of cardiac hypertrophy and impending failure [67]. Unlike the HV-MEC fetuses, HV-VIA fetuses did not show an upregulation in MYH7 and although MYH6 was numerically lower this effect was not significant. The difference in myosin isoform response between these two groups combined with the relative increase in previously discussed indicators of cardiac hypertrophy in meconium stained fetuses may suggest that susceptibility of the heart is a determinant in the loss of fetal viability.

Both maternal and fetal PRRSV infection results in significant disruption in the thyroid hormone system consistent with an NTIS-like effect. The larger decrease in T3 observed in resilient fetuses (HVL-VIA) compared to their susceptible counterparts (HVL-MEC) suggests this effect is an adaptive or protective mechanism employed by the host rather than an additional pathology subsequent to PRRSV infection. This hypothyroid state causes minimal alteration to the receptor pathway in the brain suggesting a sparing-like effect, while expression in the heart is more consistent with decompensation. This difference is manifested in the degree of disruption observed in genes associated with cell cycle progression, which was found to be significantly dysregulated in the heart. The observed responses in genes associated with cardiac function in many ways are inconsistent with what would be expected based on past research utilizing fetal thyroidectomy. These incongruities may stem from an inability to differentiate between the effect of fetal hypothyroidism and viral infection that appears to cause it. Whatever the cause, the observed alteration in cardiac gene expression within the meconium-stained fetuses is consistent with that observed with cardiac hypertrophy, which may represent a causative mechanism behind their susceptibility in the face of PRRSV infection.

## Data Availability

The datasets generated during and/or analysed during the current study are available from the corresponding author on reasonable request.

## Abbreviations

PRRSV: Porcine reproductive and respiratory virus 2 (Strain NVSL 97-7895)
NTIS: Non-thyroidal illness syndrome
Con: control fetuses collected from mock infected gilts
HV-VIA: Viable fetuses from PRRSV infected gilts with greater than 5 log at 21 dpi and 4.5 log at 12 dpi of virus in both fetal serum and thymus
HV-MEC: Meconium stained fetuses from PRRSV infected gilts with greater than 5 log at 21 dpi and 4.5 log at 12 dpi of virus in both fetal serum and thymus
UNIF: Viable fetuses from PRRSV infected gilts with no detectable virus in either fetal serum or thymus
T4: thyroxin
T3: triiodothyronine
rT3: reverse triiodothyronine
